# Relevance of Titin Missense and Non-Frameshifting Insertions/Deletions Variants in Dilated Cardiomyopathy

**DOI:** 10.1038/s41598-019-39911-x

**Published:** 2019-03-11

**Authors:** Oyediran Akinrinade, Tiina Heliö, Ronald H. Lekanne Deprez, Jan D. H. Jongbloed, Ludolf G. Boven, Maarten P. van den Berg, Yigal M. Pinto, Tero-Pekka Alastalo, Samuel Myllykangas, Karin van Spaendonck-Zwarts, J. Peter van Tintelen, Paul A. van der Zwaag, Juha Koskenvuo

**Affiliations:** 1 Children’s Hospital, Institute of Clinical Medicine, Helsinki University Central Hospital, University of Helsinki, Helsinki, Finland; 2 Institute of Biomedicine, University of Helsinki, Helsinki, Finland; 3 Heart and Lung Centre, Helsinki University Hospital and University of Helsinki, Helsinki, Finland; 4 Department of Clinical Genetics, Academic Medical Centre, University of Amsterdam, Amsterdam, The Netherlands; 5 University of Groningen, University Medical Centre Groningen, Department of Genetics, Groningen, The Netherlands; 6 Department of Cardiology, University of Groningen, University Medical Centre Groningen, Groningen, The Netherlands; 7 Department of Cardiology, Academic Medical Centre, University of Amsterdam, Amsterdam, The Netherlands; 8 Blueprint Genetics, Helsinki, Finland; 9 Durrer Centre for Cardiovascular Research, Netherlands Heart Institute, Utrecht, The Netherlands

**Keywords:** Genetic testing, Cardiovascular genetics

## Abstract

Recent advancements in next generation sequencing (NGS) technology have led to the identification of the giant sarcomere gene, titin (*TTN*), as a major human disease gene. Truncating variants of *TTN* (TTNtv) especially in the A-band region account for 20% of dilated cardiomyopathy (DCM) cases. Much attention has been focused on assessment and interpretation of TTNtv in human disease; however, missense and non-frameshifting insertions/deletions (NFS-INDELs) are difficult to assess and interpret in clinical diagnostic workflow. Targeted sequencing covering all exons of *TTN* was performed on a cohort of 530 primary DCM patients from three cardiogenetic centres across Europe. Using stringent bioinformatic filtering, twenty-nine and two rare *TTN* missense and NFS-INDELs variants predicted deleterious were identified in 6.98% and 0.38% of DCM patients, respectively. However, when compared with those identified in the largest available reference population database, no significant enrichment of such variants was identified in DCM patients. Moreover, DCM patients and reference individuals had comparable frequencies of splice-region missense variants with predicted splicing alteration. DCM patients and reference populations had comparable frequencies of rare predicted deleterious *TTN* missense variants including splice-region missense variants suggesting that these variants are not independently causative for DCM. Hence, these variants should be classified as likely benign in the clinical diagnostic workflow, although a modifier effect cannot be excluded at this stage.

## Introduction

With a prevalence of at least 1:2500, dilated cardiomyopathy (DCM) is a relatively common cause for heart failure and sudden cardiac death, and the most prevalent indication for heart transplantation^1,2^. DCM is characterized by progressive left ventricular dilatation and systolic dysfunction. Based on aetiology, the European Society of Cardiology (ESC) classified DCM into familial (genetic) and non-familial forms, with up to 30–50% of idiopathic DCM cases being familial^3,4^. Familial DCM exhibits genetic heterogeneity and follows generally an autosomal dominant inheritance. Most of the disease-causing DCM variants have been identified in genes encoding the components of the sarcomere, desmosome, cytoskeleton, nuclear lamina, mitochondria, and calcium-handling proteins^5,6^.

Variants leading to truncations of the giant sarcomeric protein titin (TTNtv) are the most frequent cause of DCM. Recently, we and others have identified clinically relevant TTNtv in about 20% of unselected and familial DCM cases^7–10^. Interestingly, DCM-causing TTNtv are clustered in the clinically relevant A-band region of the gene whereas TTNtv in reference and healthy individuals are clustered more in the I-band region where they may be spliced out, due to the usage of an alternative promoter, from mature long *TTN* isoforms resulting in minimal or no functional consequences^11^. Interpretation of TTNtv in clinical setting has been challenged by several factors including lack of knowledge of the true frequency of TTNtv in the general population, lack of understanding of different protein domains and alternatively spliced isoforms, and variable disease presentation hampering assessment of co-segregation. We and others have previously addressed these issues^7,9,12–14^. However, assessing the relevance of *TTN* missense variants in the pathogenesis of DCM has been even more difficult due to the high frequency of these variants in both reference individuals and DCM patients and the absence of large pedigrees where segregation can be studied. While few studies have reported modifier roles for *TTN* missense variants^15,16^, others have considered *TTN* missense variants as disease-causing variants in DCM, arrhythmogenic right ventricular cardiomyopathy (ARVC), and left ventricular non-compaction cardiomyopathy (LVNC) but these observations suggest recessive inheritance pattern and no convincing segregation has been demonstrated so far^17–19^. Furthermore, TTNtvs are also occasionally found in patients with SCD/aborted SCD who do not have diagnosis of DCM or DCM with LVNC^20,21^

In the current study, we focused on *TTN* missense and non-frameshifting insertion-deletion (NFS-INDELs) variants identified in DCM patients and reference population. By analyzing *TTN* missense and NFS-INDELs variants identified in 530 primary DCM patients, over 60,000 ExAC individuals, and for the first time, the largest available 123,136 gnomAD database reference individuals, we report a seemingly insignificant enrichment of *TTN* missense variants in DCM patients compared with reference individuals.

## Results

### Patient Cohort

DCM patients in this multi-centre study were recruited from three major cardiogenetic centres in Finland and the Netherlands. All patients fulfilled diagnostic criteria for DCM. Table 1 summarizes spectrum of pathogenic and likely pathogenic variants identified in the patients.

**Table 1:** Distribution and spectrum of other pathogenic and likely pathogenic variants identified in the cohort.

Patients	All (N = 530)	Helsinki cohort (N = 145)	Groningen cohort (N = 169)	Amsterdam cohort (N = 216)
***Gender distribution, n (%)***				
Males	311 (58.7)	107 (73.8)	95 (56.2)	109 (50.5)
Females	219 (41.3)	38 (26.2)	74 (43.8)	107 (49.5)
***Diagnostic yield, n (%)***				
Mutation positive	157 (29.6)	51 (35.2)	37 (21.9)	69 (31.9)
Mutation negative	373 (70.4)	94 (64.8)	137 (81.1)	147 (68.1)
***Causative gene, n (%)***				
**Sarcomeric**	**108 (20.4)**	**28 (19.3)**	**22 (13.0)**	**58 (26.9)**
*TPM1*	1 (0.2)	—	—	1 (0.5)
*TCAP*	1 (0.2)	1 (0.7)	—	—
*TNNT2*	2 (0.4)	1 (0.7)	—	1 (0.5)
*TNNI3*	2 (0.4)	—	—	2 (0.9)
*MYH7*	5 (0.9)	1 (0.7)	2 (1.2)	2 (0.9)
*TTN*	97 (18.3)	25 (17.2)	20 (11.8)	52 (24.1)
**Non-sarcomeric**	**49 (9.2)**	**23 (15.7)**	**15 (8.9)**	**11 (5.1)**
*DMD*	1 (0.2)	1 (0.7)	—	—
*RBM20*	10 (1.9)	2 (1.4)	3 (1.8)	5 (2.3)
*PLN*	11 (2.1)	—	7 (4.1)	4 (1.9)
*DSP*	12 (2.3)	8 (5.5)	2 (1.2)	2 (0.9)
*LMNA*	15 (2.8)	12 (8.3)	3 (1.8)	—

### Deriving Allele Frequency for Potentially Deleterious DCM Variant

To set a threshold for filtering *TTN* missense variants identified in DCM cohorts, we obtained the gnomAD allele frequency of the most common pathogenic variant associated with DCM with a wide consensus of the pathogenicity in the field. Covering 100% of the total gnomAD alleles, the *PLN* c.40_42delAGA, p.Arg14del (NM_002667.4) variant is the most common “certainly pathogenic” DCM variant^22–26^. This variant is absent in the ExAC individuals (allele count [AC] = 0), while it is present in two individuals (AC = 2) in the gnomAD database. Based on these observations, the minor allele frequency (MAF) cut-offs were set to 0.0016% (AC = 2) and 0.0016% (AC = 4) for ExAC and gnomAD datasets respectively, and variants with higher frequency in the reference populations would not be expected to be pathogenic.

### *TTN* NFS-INDELs and Missense Variants in DCM Cohort

**Figure 1:**
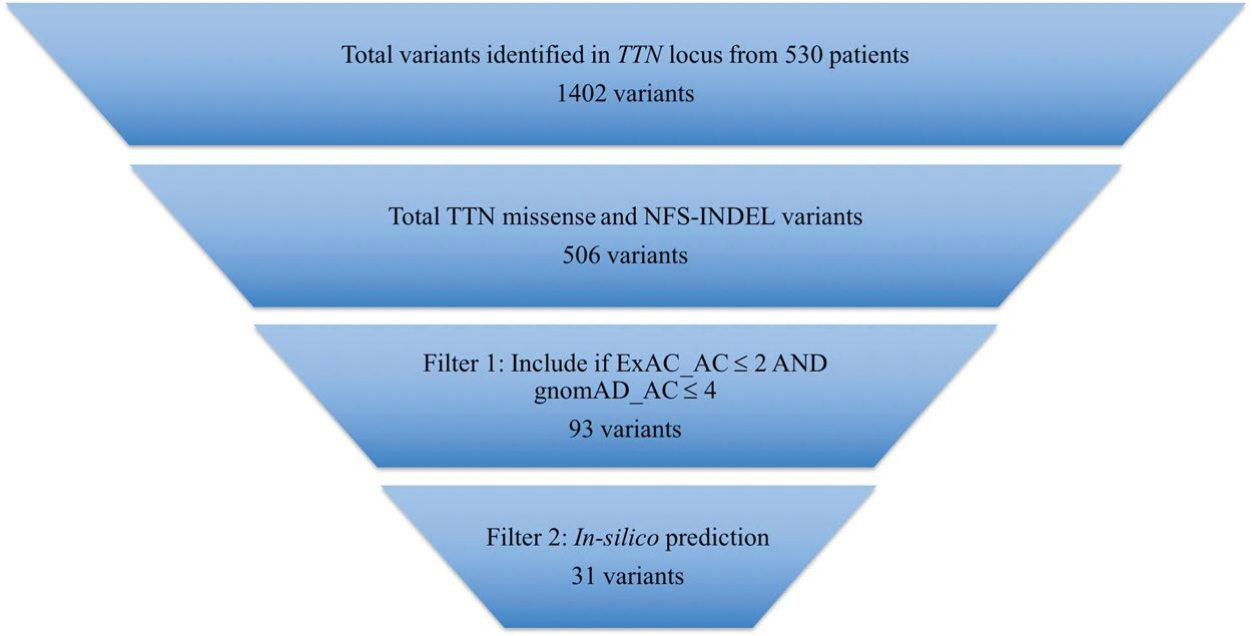
Bioinformatic filtering strategy to identify “deleterious” variants. Total *TTN* variants were filtered using allele frequency (0.0016%) threshold derived from the frequency of known pathogenic DCM variants identified in gnomAD reference individuals. Variants more frequent than derived thresholds in respective sources were not expected to be deleterious.

**Figure 2:**
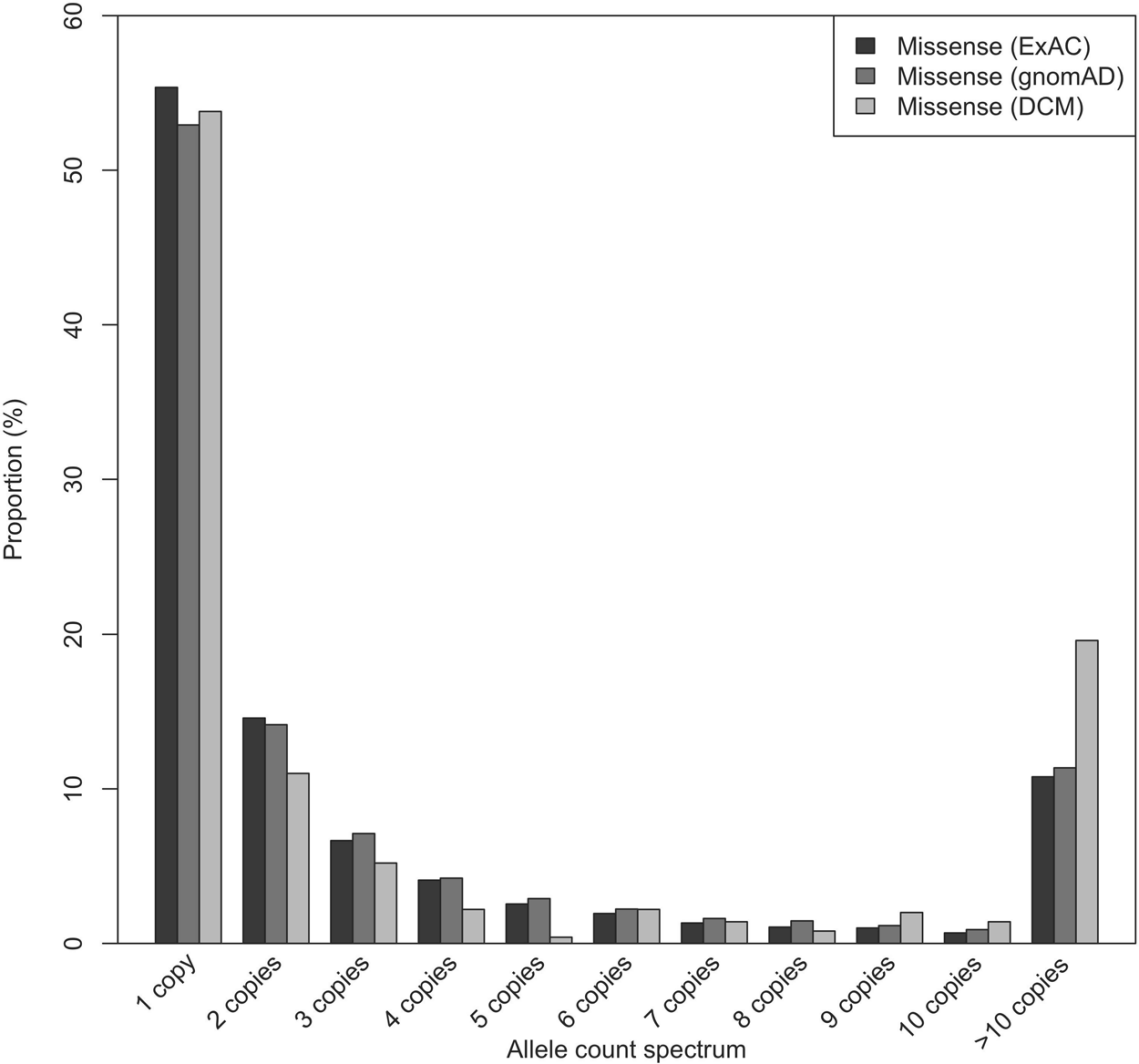
Allele frequency spectrum of *TTN* missense variants in DCM and reference individuals. More than 50% of *TTN* missense variants in DCM patients as well as in reference individuals were private. Potentially pathogenic private and/or rare *TTN* missense variants were not enriched in DCM patients compared with ExAC and gnomAD reference individuals.

Initial variant calling with GATK yielded a total of 1402 unique *TTN* variants identified in 530 primary DCM patients, of which 506 were missense and NFS-INDELs (Fig. 1), 39 frameshift variants, 27 nonsense variants, 14 splice-site variants, 205 silent variants, 4 untranslated region (UTR) variants, and the remaining 607 variants being intronic. Of the total variants, 52.7% were private (Fig. 2). Applying the ExAC and gnomAD MAF cut-offs yielded a total of 87 missense and 5 NFS-INDEL variants. Taken together, 18.9% and 1.9% of DCM patients carried rare heterozygous missense and NFS-INDELs, respectively. Given that many rare and even private variants are believed to be innocent bystanders, variant pathogenicity potential was further assessed by using three *in-silico* prediction tools. Implementing this, 33.3% (29/87) of the rare missense variants were predicted deleterious. Interestingly, 69.0% (20/29) of the predicted deleterious missense variants were in the A-band region of *TTN*. Of note, 55.2% (16/29) of the rare missense variants were absent in both ExAC and gnomAD reference individuals.

Twenty-nine rare heterozygous *TTN* missense variants predicted deleterious were identified in 6.8% (36/530) of DCM patients, with only one patient harbouring two rare heterozygous *TTN* missense variants. Heterozygous NFS-INDELS affecting at least five transcripts of the *TTN* (Transcript Count Index, TCI ≥ 5) were identified in 0.37% (2/530) of DCM patients. Of note, all the NFS-INDELS were in the I-band region of *TTN* (Tables 2 and 3). When considering mutation status, gene elusive DCM patients and mutation positive DCM patients had comparable frequencies of rare *TTN* missense variants further suggesting that these variants may not be monogenic cause of DCM (Supplemantary Table S1).

**Table 2:** *TTN* missense and non-frameshifting INDELs variants in DCM vs. general population.

	Total *TTN* NFS-INDEL allele				Total *TTN* missense allele			
	DCM (n = 530)	gnomAD (n = 123,136)	OR	p-value	DCM (n = 530)	gnomAD (n = 123,136)	OR	p-value
***Raw variant (before assessment)***								
Unique variant count	9	289			497	17726		
***After assessment using derived thresholds***								
Unique variant count	5	226			87	13808		
Number of individuals (n)	10	337			100	21102		
Prevalence (%)	1.887	0.273	7.0	3.2E-06	18.867	17.137	1.1	0.3187
**Distribution by sarcomere domain, n (%)**								
Z-disc	0 (0.00)	32 (0.026)		NA	2 (0.377)	1074 (0.872)	0.4	0.3400
I-band	10 (1.887)	104 (0.084)	22.7	8.7E-11	46 (8.679)	6720 (5.457)	1.6	0.0015
A-band	0 (0.00)	137 (0.111)		NA	58 (10.943)	10725 (8.709)	1.3	0.0815
M-band	0 (0.00)	53 (0.043)		NA	2 (0.377)	1488 (1.208)	0.3	0.1000
***After assessment using derived thresholds and pathogenicity prediction***								
Unique variant count	2	172			29	4657		
Number of individuals (n)	2	255			37	7071		
Prevalence (%)	0.377	0.207	1.8	0.3000	6.981	5.74	1.2	0.2200
**Distribution by sarcomere domain, n (%)**								
Z-disc	0 (0.00)	31 (0.025)		NA	1 (0.188)	243 (0.197)	0.95	1.0000
I-band	2 (0.377)	30 (0.024)	15.5	0.0083	10 (1.887)	1486 (1.206)	1.6	0.1581
A-band	0 (0.00)	137 (0.111)		NA	25 (4.717)	4760 (3.865)	1.2	0.3100
M-band	0 (0.00)	53 (0.043)		NA	0	540 (0.438)	NA	NA

**Table 3:** Rare *TTN* missense variant classified as deleterious by *in-silico* prediction.

SYMBOL	Position	Nucleotide Change	AA Change	EXON	DCM (n)	ExAC (n)	gnomAD (n)
Z-disk							
*TTN*	179664242	c.886 G > A	Val296Met	6	1	0	0
I-band							
*TTN*	179640476	c.6115 C > T	Leu2039Phe	28	1	0	0
*TTN*	179634837	c.8591 A > G	Tyr2864Cys	36	1	0	0
*TTN*	179634555	c.8753 G > C	Gly2918Ala	37	1	1	2
*TTN*	179632835	c.9211 G > C	Glu3071Gln	39	1	2	4
*TTN*	179588312	c.21515 G > T	Arg7172Ile	74	3	0	0
*TTN*	179576946	c.27611 C > T	Pro9204Leu	96	1	1	2
*TTN*	179574299	c.28747 A > T	Ile9583Phe	99	1	0	0
*TTN*	179494979	c.44270 T > A	Leu14757His	239	2	0	0
A-band							
*TTN*	179477634	c.49814 T > G	Val16605Gly	265	6	0	0
*TTN*	179476201	c.50755 G > T	Val16919Phe	269	1	0	0
*TTN*	179472191	c.53224 T > A	Tyr17742Asn	277	1	0	0
*TTN*	179452778	c.63356 G > A	Cys21119Tyr	305	1	1	1
*TTN*	179441303	c.69668 G > C	Gly23223Ala	325	1	0	0
*TTN*	179425479	c.85380 A > G	Ile28460Met	326	1	1	3
*TTN*	179429090	c.81769 C > A	Pro27257Thr	326	0	0	0
*TTN*	179432751	c.78108 G > C	Leu26036Phe	326	1	0	1
*TTN*	179435376	c.75483 G > C	Lys25161Asn	326	1	0	1
*TTN*	179436260	c.74599 A > G	Thr24867Ala	326	1	0	0
*TTN*	179439256	c.71603 G > A	Arg23868Gln	326	1	1	2
*TTN*	179439440	c.71419 T > C	Tyr23807His	326	1	0	0
*TTN*	179419452	c.88622 T > C	Ile29541Thr	332	1	1	4
*TTN*	179416971	c.90656 T > A	Val30219Asp	335	1	0	0
*TTN*	179417593	c.90034 G > A	Glu30012Lys	335	1	0	0
*TTN*	179418010	c.89617 T > C	Trp29873Arg	335	1	0	0
*TTN*	179414844	c.91721 A > T	Glu30574Val	337	2	0	1
*TTN*	179414390	c.92059 G > A	Gly30687Ser	338	1	2	4
*TTN*	179411891	c.94361 T > C	Leu31454Pro	340	1	0	0
*TTN*	179403949	c.98713 G > A	Glu32905Lys	353	1	1	3

Variant positions were reported for the RefSeq transcript NM_001267550.

### *TTN* Missense and NFS-INDELs Variants in Reference Population

Applying the derived allele frequency thresholds to *TTN* variants reported in ExAC, we identified 8265 and 82 rare missense and NFS-INDELs variants respectively in 16.5% (10035/60707) and 0.15% (93/60707) of the ExAC individuals. Of the ExAC rare missense variants, 24% (1990/8265) were predicted deleterious by the three prediction tools used in this study. In total, 3.98% of ExAC individuals carried a rare *TTN* missense variant predicted deleterious. In addition, 0.11% (65/60706) of ExAC cohort harboured rare heterozygous NFS-INDELs affecting at least five transcripts of *TTN*.

Given that gnomAD cohort is almost twice the size of ExAC cohort and ExAC individuals are in gnomAD cohort, we set the threshold to four and considered variants more frequent than this in gnomAD as likely benign. With this threshold, we identified 13808 rare missense variants present in 17.1% (21102/123136) of gnomAD cohort. However, rare heterozygous *TTN* missense variants predicted deleterious were observed in 5.7% (7071/123136) of the gnomAD (Table 2). Notably, 54.6% and 52.2% of *TTN* missense and NFS-INDELS identified in ExAC and gnomAD cohorts respectively were private (Fig. 2).

### Multiple Heterozygous *TTN* Variants

To estimate the frequency of reference individuals with multiple heterozygous *TTN* variants, the phase 3 call set of the 1000 Genomes project was utilized since it provides sample-level genotype data. Only *TTN* missense variants with allele frequency less than or equal the derived thresholds were used for this analysis. We identified 8.2% (205/2504) of the 1000 Genomes Project phase 3 cohorts with a rare heterozygous *TTN* missense variant. Of these, 5.4% (11/205) harboured two heterozygous rare *TTN* missense variants.

When considering eleven individuals in 1000 Genomes Project phase 3 cohorts with *TTN* truncations (TTNtv) as published previously^12^, 18.2% (2/11) of these individuals, both from South Asian population also harboured a rare heterozygous *TTN* missense variant, thus, being heterozygous for both rare TTNtv and *TTN* missense variant. However, it is unclear whether they are in *cis* (at the same allele) or in *trans* (at different allele)

Interestingly, only one DCM patient in this study harboured two rare heterozygous *TTN* missense variants, one in the A-band and the other at the distal I-band *TTN* region. Five out of thirty-six (13.9%) DCM patients with rare *TTN* missense variants predicted deleterious harbored a TTNtv (Table 4).

**Table 4:** DCM patients with both predicted deleterious *TTN* missense variant and *TTN* truncation (TTNtv).

Gene	Deleterious *TTN* missense variants		Other known P/LP TTNtv	
	Nucleotide Change	AA Change	Nucleotide Change	AA Change
*TTN*	c.44270 T > A	p.Leu14757His	c.45322 C > T	p.Arg15108*
*TTN*	c.91721 A > T	p.Glu30574Val	c.76373delC	p.Pro25458Glnfs*9
*TTN*	c.98713 G > A	p.Glu32905Lys	c.8426dup	p.Asn2809Lysfs*7
*TTN*	c.69668 G > C	p.Gly23223Ala	c.69671delC	p.Pro23224HisfsTer10
*TTN*	c.49814 T > G	p.Val16605Gly	c.75391delG	p.V25131Lfs*16

Variant positions were reported for the RefSeq transcript NM_001267550.

### Distribution of *TTN* NFS-INDELs and Missense Variants in DCM and Reference Cohorts

**Figure 3:**
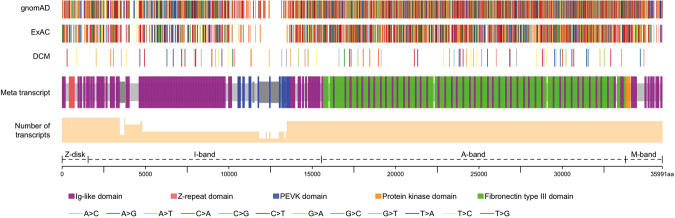
Spatial distribution of rare *TTN* missense variants in DCM cohort and reference population. Titin is linearly depicted with its 152 Ig-like domains in purple and 132 fibronectin type III domains in green. *TTN* missense variants identified in patients as well as ExAC and gnomAD reference individuals are shown as coloured bars. Bisque bar represents the number of transcripts (TCI). Variants are shown relative to the Meta transcript position. The dark grey bars indicate exons unique to the Meta transcript. The dashed lines below the number of transcripts schematic indicate the location of variants within the sarcomere.

The proportion of DCM patients harbouring rare *TTN* missense and NFS-INDEL variants before and after variant assessment was compared with that identified in the reference population. 18.9% (100/530) of DCM patients harboured rare *TTN* missense variants as against 17.3% in gnomAD reference individuals (OR = 1.1; 95% CI: 0.90–1.35; *P* = 0.32). Moreover, DCM patients had a slightly higher frequency of *TTN* missense in the *TTN* I-band region (8.7% vs. 5.5%; OR = 1.1; 95% CI: 1.2–2.1; *P* = 0.0015) and A-band region (10.9% vs. 8.7%; OR = 1.3; 95% CI: 1.0–1.6; *P* = 0.08). Rare NFS-INDELs was detected in 1.9% of DCM patients as against 0.3% in gnomAD reference individuals (OR = 7.0; *P* = 3.2 × 10^−6^). Of note, all NFS-INDELs identified in DCM patients clustered in the *TTN* I-band region. When considering rare *TTN* missense and NFS-INDELs together, there was no statistically significant enrichment of these variants in DCM patients and within *TTN* domains compared to the observed frequency and distribution in gnomAD database reference individuals (Table 2 and Fig. 3). Moreover, distribution of *TTN* missense variants was similar in patients with a known pathogenic or likely pathogenic DCM mutation (Mutation positive) compared to those without a mutation (Mutation negative) when the variant classification follows ACMG criteria (Supplementary Table S1).

### Splice-Region Missense Variants with Predicted Splicing Effect are not enriched in DCM

Given that variants that affect splicing can directly cause disease or contribute to disease severity, we analysed missense variants located near exon-intron boundaries (splice-regions). A total of five rare splice-region missense variants affecting 3.6% (19/530) of DCM patients was identified. Of note, only two out of five were predicted to potentially alter natural splice site or activate exonic cryptic splice-sites. When compared with ExAC reference individuals (DCM: 3.6% vs. ExAC: 2.9%; *P* = 0.4624) and gnomAD reference individuals (DCM: 3.6% vs. gnomAD: 3.0%; *P* = 0.4808), there was no significant enrichment of variants with high potential for defective splicing in DCM patients.

## Discussion

Recent advancement in high-throughput sequencing technology has unravelled *TTN* as a major human disease gene, mutated in both human skeletal and cardiac muscles disease. Inclusion of the *TTN* gene in genetic screening for DCM has led to an increase in diagnostic rate for adult DCM by at least 18% and 25% respectively in sporadic and familial DCM cases^8–10,27,28^. Much attention has been focused on the assessment and clinical interpretation of frameshift, nonsense and splice-site variants, leading to a truncation of *TTN*, leaving missense and NFS-INDELs variants in this major human disease gene unassessed and unexplored. Consequently, these variants are difficult to assess and interpret in the clinical diagnostic workflow.

DCM is a genetically heterogeneous disorder and multiple genes have been implicated in the pathogenesis of DCM. Truncations of the giant sarcomeric protein, *TTN*, are to date, the most common genetic cause of DCM, accounting for about 20% of cases, and are overrepresented in the A-band region of the protein. Such A-band and/or distal I-band TTNtv have been estimated to have nearly 98% chance of being disease causing when identified in unselected DCM patients^7^. Interestingly, a recent study showed a significant association of TTNtv in constitutive exons throughout *TTN* protein with DCM^29^. Furthermore, it has been shown that TTNtv rarely cause pediatric DCM but the gene typically expresses the disease at middle-age^30^. In addition, we and others have addressed several issues and challenges associated with the clinical assessment and interpretation of TTNtv in both research and clinical diagnostic setting^7,9,12–14^. In contrast, the role and contribution of missense and NFS-INDELs variants in this gene has been poorly addressed so far.

In this European multi-centre DCM study, we assessed the possible role of *TTN* missense and NFS-INDELs variants in DCM. By analyzing *TTN* variants in: 1) a large primary DCM cohort (n = 530), 2) the widely used ExAC reference individuals, and 3) the largest available gnomAD database reference individuals, we showed that there is a statistically insignificant enrichment of rare *TTN* missense and NFS-INDEL variants in DCM patients compared with reference individuals. This observation holds even when considering different *TTN* regions, suggesting that *TTN* missense and NFS-INDEL variants do not cause DCM and should therefore be classified as “likely benign” in clinical diagnostic settings. Using stringent bioinformatic filtering criteria, we identified rare *TTN* missense variants predicted deleterious in 7.0% of primary DCM cases as against 5.7% in gnomAD reference individuals (OR = 1.2; *P* = 0.22). When considering different *TTN* domains (Z-disk, I-band, A-band and M-band), we identified no enrichment of these variants in DCM patients compared to reference individuals.

A recent study assessing the role of *TTN* missense variants identified “severe” missense variants in 25.2% (37/147) of DCM probands^15^. We randomly selected five “severe” missense variants as published by the authors and applied our bioinformatic filtering strategy. Of note, 40% (2/5) of these “severe” variants were found in at least seven gnomAD database reference individuals, and as such would not be considered “deleterious” using our approach. Interestingly, five probands in the study were double heterozygous for a “severe” *TTN* missense variant and a pathogenic (P)/likely pathogenic (LP) variant in *LMNA, MYH7* and *SCN5A*. Three (3/5) of these patients harboured a P/LP *LMNA* variant in addition to “severe” *TTN* variant. Two of the “severe” *TTN* missense variants were later reclassified by the authors as “unlikely” disease associated, possibly due to lack of segregation, and third patient harbouring a P/LP *LMNA* variant co-occurring with a “severe” *TTN* missense variant had undergone heart transplantation^15^. In another study of an extended DCM family with 14 affected subjects, four had severe DCM requiring heart transplantation in early adulthood^31^. In all affected patients, the P/LP *LMNA* p.(Lys219Thr) variant was identified. In addition to this variant, a *TTN* missense variant, p.(Leu4855Phe), was identified in all four patients with severe phenotypes, all of whom had transplantation at a younger age compared to those with only *LMNA* p.(Lys219Thr) variant. Although this may suggest that *TTN* missense variants can modify disease severity, the *TTN* p.(Leu4855Phe) is found heterozygous in 15 individuals in gnomAD reference population and the position is multi-allelic, thus stronger evidence is needed before scientific interpretation of potential modifier role can be inferred.

Disruption of natural splice sites by point mutations can result in exon skipping, activation of cryptic splice-site, and rarely intron retention^32^, with activation of cryptic splice-site being the second most frequent consequence of such mutations. Such point mutations, put together, account for 10–15% of all mutations causing human inherited disease^33,34^. By analyzing missense variants located near exon-intron boundaries (splice-region) of *TTN*, we identified five rare splice-region missense variants in DCM patients. Two (40%) of these five splice-region missense variants were predicted to disrupt natural splice-site. To test for possible enrichment of this event in DCM patients, we randomly and iteratively selected five rare splice-regions variants from gnomAD and tested their potential to alter splicing. Surprisingly, two (40%) of the five randomly chosen splice-region missense variants were predicted to alter splicing, suggesting that splice-region missense variants identified in our DCM cohort do not carry higher risk of splicing defect than any other splice-region missense variants in the population.

DCM cohorts in this study were sequenced using high coverage sequencing assays yielding more uniform coverage even across difficult-to-sequence regions that may alone increase likelihood of finding slightly higher variant counts from DCM cohorts compared to the population databases. If we overestimate that DCM has a prevalence of 1 per 500 and further speculate that *TTN* missense variants would account 10% of all DCM cases leading to a frequency of 1 per 5000 (0.02%) in *TTN* missense positive DCM. However, 5.7% of gnomAD reference population have rare missense variant predicted deleterious by *in-silico* tools, thus 99.7% of these missense variants are not expected to cause DCM in monogenic manner. Thus, these variants should be classified as likely benign in clinical and diagnostic workflow as far as more understanding have been gathered of them individually and as a group.

Rare NFS-INDELs affecting at least five transcripts of *TTN* (TCI ≥ 5) were identified in 0.37% (2/530) of DCM patients as against 0.21% (255/123136) in gnomAD reference individuals (OR = 1.8; *P* = 0.3). Failure to show significant enrichment of NFS-INDELs in our DCM cohort suggests that the vast majority of these variants are non-contributory to the monogenic etiology of DCM.

### Limitations

We acknowledge the following limitations: The health status of reference individuals used is not known even though curators of reference population databases have made every effort to exclude individuals with severe pediatric diseases from these cohorts. Moreover, we had limited evidence of the effect of predicted deleterious *TTN* missense variants in patients with other P/LP variants including TTNtv. Functional analysis of predicted deleterious TTN missense and NFS-INDELs was beyond the scope of this study; and we recommend that such should be combined with segregation information and de novo status to reclassify the variants.

## Conclusion

The current data revealed that predicted deleterious *TTN* missense and NFS-INDEL variants are not significantly enriched in a large group of DCM patients compared to control populations, and should therefore be classified as likely benign in a clinical diagnostic setting. Nevertheless, it cannot be excluded that some rare *TTN* missense variants might have modifier effect for the phenotype.

## Methods

### Patient Cohorts and Clinical Evaluation

This European multi-centre study was approved by institutional ethical committees of the University of Helsinki, Academic Medical Centre (Amsterdam, the Netherlands), and University Medical Centre (Groningen, the Netherlands) and informed consent was obtained from all subjects. The DCM cohort in this study comprised of 530 unrelated primary DCM patients of European origin. The patients were recruited at three major cardiogenetic centres in Finland (Helsinki; n = 145) and the Netherlands (Amsterdam; n = 216, and Groningen; n = 169). All patients fulfilled the diagnostic criteria for DCM (LV size/volume >117% or >2 SD of the reference value and LV-EF <45%)^35^.

### Genetic Studies of DCM

Genomic DNA was extracted from patients’ blood samples by standard procedures and guidelines. *TTN* was sequenced in a total of 530 primary DCM patients using targeted panels of increasing sizes as previously described^8,27^. Irrespective of the sequencing panel used, sequence data from each patient was processed uniformly following GATK best practices recommendation to identify genetic variants^36^. Identified variants were annotated using Ensembl’s Variant Effect Predictor (VEP v87) tool^37^, and SIFT^38^, PolyPhen^39^, and MutationTaster^40^ pathogenicity scores and predictions were obtained for each missense variant via the database of nonsynonymous SNP functional prediction (dbNSFPv2.9.2)^41^. Rare *TTN* missense variants predicted deleterious by the three prediction tools were considered candidate risk variants. NFS-INDELs were assessed using the Transcript Count Index (TCI) as previously published^7^.

### Analysis of *TTN* Missense and NFS-INDELs Variants in the Population

Exonic boundaries of *TTN* in build 37 of the human genome (hg19) were downloaded from the Ensembl Genome Browser (www.ensembl.org), and were used to query ExAC and gnomAD databases (accessed 23 July 2017) for *TTN* exonic variants. Extracted variants were filtered for quality (Phred Quality Score 29) and coverage (15.0X), and calls that passed all quality filters were used for downstream analysis. The two datasets were re-annotated using VEP v87, processed uniformly to identify spectrum and distribution of missense and NFS-INDELS mutations in the reference population.

### Variant Mapping to Uniprot Protein Domain

To allow inclusion of variants located in exons not present in the principal cardiac long isoform (N2BA), otherwise known as the canonical transcript with the Uniprot ID Q8WZ42-1, the location of variants identified in DCM patients as well as in the reference population was reported with respect to the Meta transcript annotation - (ENST00000589042/NM_001267550). Protein domain annotation for the Uniprot consensus sequence Q8WZ42-1 was transferred to the inferred Meta transcript as described earlier^7,12^.

### *In-silico* Splicing Defect Prediction

The effect of splice-region missense variants on splicing efficiency were predicted with five different tools – Human Splicing Finder^42,43^, Splice Site Prediction by Neural Network (NNSPLICE)^44^, MaxEntScan^45^, GeneSplicer^46^, and Splice Site Finder, via Alamut splicing software v2.0 (Interactive Biosoftware, France) using default settings in all predictions. Controls for each rare splice-region missense variant were selected from gnomAD from near-by genomic location and with similar allele frequency.

### Statistical Analysis

Between groups comparisons for categorical variables were performed using χ^2^ test if appropriate, otherwise, Fisher’s exact test. For non-parametric and continuous variables, between groups comparisons were done using Mann–Whitney *U* tests and independent samples *t*-tests combined with Levene’s tests respectively. Bonferroni correction was performed on all analyses to adjust for multiple testing. All statistical analyses were done using R statistical software (version 3.3.3).

## Supplementary information

Supplementary File

## References

[1] Codd MB, Sugrue DD, Gersh BJ, Melton LJ (1989). Epidemiology of idiopathic dilated and hypertrophic cardiomyopathy. A population-based study in Olmsted County, Minnesota, 1975–1984. Circulation.

[2] John R (2001). Long-term outcomes after cardiac transplantation: an experience based on different eras of immunosuppressive therapy. The Annals of thoracic surgery.

[3] Elliott P (2008). Classification of the cardiomyopathies: a position statement from the European Society Of Cardiology Working Group on Myocardial and Pericardial Diseases. European heart journal.

[4] Towbin JA, Bowles NE (2002). The failing heart. Nature.

[5] Ahmad F, Seidman JG, Seidman CE (2005). The genetic basis for cardiac remodeling. Annual review of genomics and human genetics.

[6] Dellefave L, McNally EM (2010). The genetics of dilated cardiomyopathy. Current opinion in cardiology.

[7] Akinrinade O, Alastalo TP, Koskenvuo JW (2016). Relevance of truncating titin mutations in dilated cardiomyopathy. Clin Genet.

[8] Akinrinade O (2015). Genetics and genotype-phenotype correlations in Finnish patients with dilated cardiomyopathy. European heart journal.

[9] Herman DS (2012). Truncations of titin causing dilated cardiomyopathy. The New England journal of medicine.

[10] Pugh TJ (2014). The landscape of genetic variation in dilated cardiomyopathy as surveyed by clinical DNA sequencing. Genetics in medicine: official journal of the American College of Medical Genetics.

[11] Deo RC (2016). Alternative Splicing, Internal Promoter, Nonsense-Mediated Decay, or All Three: Explaining the Distribution of Truncation Variants in Titin. Circulation. Cardiovascular genetics.

[12] Akinrinade O, Koskenvuo JW, Alastalo TP (2015). Prevalence of Titin Truncating Variants in General Population. PloS one.

[13] Jansweijer JA (2017). Truncating titin mutations are associated with a mild and treatable form of dilated cardiomyopathy. European journal of heart failure.

[14] van Spaendonck-Zwarts KY (2014). Titin gene mutations are common in families with both peripartum cardiomyopathy and dilated cardiomyopathy. European heart journal.

[15] Begay, R. L. *et al*. Role of Titin Missense Variants in Dilated Cardiomyopathy. *Journal of the American Heart Association* **4**, 10.1161/JAHA.115.002645 (2015). 10.1161/JAHA.115.002645 PMC4845231 26567375

[16] Golbus JR (2012). Population-based variation in cardiomyopathy genes. Circulation. Cardiovascular genetics.

[17] Hinson JT (2015). HEART DISEASE. Titin mutations in iPS cells define sarcomere insufficiency as a cause of dilated cardiomyopathy. Science.

[18] Hastings R (2016). Combination of Whole Genome Sequencing, Linkage, and Functional Studies Implicates a Missense Mutation in Titin as a Cause of Autosomal Dominant Cardiomyopathy With Features of Left Ventricular Noncompaction. Circulation. Cardiovascular genetics.

[19] Taylor M (2011). Genetic variation in titin in arrhythmogenic right ventricular cardiomyopathy-overlap syndromes. Circulation.

[20] Tayal U (2017). Truncating Variants in Titin Independently Predict Early Arrhythmias in Patients With Dilated Cardiomyopathy. Journal of the American College of Cardiology.

[21] Kapoor A (2016). Rare coding TTN variants are associated with electrocardiographic QT interval in the general population. Scientific reports.

[22] Walsh R (2017). Reassessment of Mendelian gene pathogenicity using 7,855 cardiomyopathy cases and 60,706 reference samples. Genetics in medicine: official journal of the American College of Medical Genetics.

[23] DeWitt MM, MacLeod HM, Soliven B, McNally EM (2006). Phospholamban R14 deletion results in late-onset, mild, hereditary dilated cardiomyopathy. Journal of the American College of Cardiology.

[24] Haghighi K (2006). A mutation in the human phospholamban gene, deleting arginine 14, results in lethal, hereditary cardiomyopathy. Proceedings of the National Academy of Sciences of the United States of America.

[25] van der Zwaag PA (2012). Phospholamban R14del mutation in patients diagnosed with dilated cardiomyopathy or arrhythmogenic right ventricular cardiomyopathy: evidence supporting the concept of arrhythmogenic cardiomyopathy. European journal of heart failure.

[26] van der Zwaag PA (2013). Recurrent and founder mutations in the Netherlands-Phospholamban p.Arg14del mutation causes arrhythmogenic cardiomyopathy. Netherlands heart journal: monthly journal of the Netherlands Society of Cardiology and the Netherlands Heart Foundation.

[27] Haas, J. *et al*. Atlas of the clinical genetics of human dilated cardiomyopathy. *European heart journal*, 10.1093/eurheartj/ehu301 (2014). 10.1093/eurheartj/ehu301 25163546

[28] Roberts AM (2015). Integrated allelic, transcriptional, and phenomic dissection of the cardiac effects of titin truncations in health and disease. Science translational medicine.

[29] Schafer S (2017). Titin-truncating variants affect heart function in disease cohorts and the general population. Nature genetics.

[30] Fatkin D (2016). Titin truncating mutations: A rare cause of dilated cardiomyopathy in the young. Progress in pediatric cardiology.

[31] Roncarati R (2013). Doubly heterozygous LMNA and TTN mutations revealed by exome sequencing in a severe form of dilated cardiomyopathy. European journal of human genetics: EJHG.

[32] Nakai K, Sakamoto H (1994). Construction of a novel database containing aberrant splicing mutations of mammalian genes. Gene.

[33] Krawczak M (2007). Single base-pair substitutions in exon-intron junctions of human genes: nature, distribution, and consequences for mRNA splicing. Hum Mutat.

[34] Krawczak M, Reiss J, Cooper DN (1992). The mutational spectrum of single base-pair substitutions in mRNA splice junctions of human genes: causes and consequences. Human genetics.

[35] Mestroni L (1999). Guidelines for the study of familial dilated cardiomyopathies. Collaborative Research Group of the European Human and Capital Mobility Project on Familial Dilated Cardiomyopathy. European heart journal.

[36] DePristo MA (2011). A framework for variation discovery and genotyping using next-generation DNA sequencing data. Nature genetics.

[37] McLaren W (2010). Deriving the consequences of genomic variants with the Ensembl API and SNP Effect Predictor. Bioinformatics.

[38] Ng PC, Henikoff S (2003). SIFT: Predicting amino acid changes that affect protein function. Nucleic acids research.

[39] Adzhubei, I., Jordan, D. M. & Sunyaev, S. R. Predicting functional effect of human missense mutations using PolyPhen-2. *Current protocols in human genetics/editorial board, Jonathan L. Haines…* [*et al*.] Chapter 7, Unit720, 10.1002/0471142905.hg0720s76 (2013). 10.1002/0471142905.hg0720s76 PMC4480630 23315928

[40] Schwarz JM, Cooper DN, Schuelke M, Seelow D (2014). MutationTaster2: mutation prediction for the deep-sequencing age. Nature methods.

[41] Liu X, Jian X, Boerwinkle E (2013). dbNSFPv2.0: a database of human non-synonymous SNVs and their functional predictions and annotations. Hum Mutat.

[42] Desmet FO (2009). Human Splicing Finder: an online bioinformatics tool to predict splicing signals. Nucleic Acids Res.

[43] Hubbard TJ (2007). Ensembl 2007. Nucleic acids research.

[44] Reese MG, Eeckman FH, Kulp D, Haussler D (1997). Improved splice site detection in Genie. J Comput Biol.

[45] Eng L (2004). Nonclassical splicing mutations in the coding and noncoding regions of the ATM Gene: maximum entropy estimates of splice junction strengths. Hum Mutat.

[46] Pertea M, Lin X, Salzberg SL (2001). GeneSplicer: a new computational method for splice site prediction. Nucleic Acids Res.

