# Publisher Correction: Relevance of Titin Missense and Non-Frameshifting Insertions/Deletions Variants in Dilated Cardiomyopathy

**DOI:** 10.1038/s41598-020-73763-0

**Published:** 2020-10-09

**Authors:** Oyediran Akinrinade, Tiina Heliö, Ronald H. Lekanne Deprez, Jan D. H. Jongbloed, Ludolf G. Boven, Maarten P. van den Berg, Yigal M. Pinto, Tero-Pekka Alastalo, Samuel Myllykangas, Karin van Spaendonck-Zwarts, J. Peter van Tintelen, Paul A. van der Zwaag, Juha Koskenvuo

**Affiliations:** 1 Children’s Hospital, Institute of Clinical Medicine, Helsinki University Central Hospital, University of Helsinki, Helsinki, Finland; 2 Institute of Biomedicine, University of Helsinki, Helsinki, Finland; 3 Heart and Lung Centre, Helsinki University Hospital and University of Helsinki, Helsinki, Finland; 4 Department of Clinical Genetics, Academic Medical Centre, University of Amsterdam, Amsterdam, The Netherlands; 5 University of Groningen, University Medical Centre Groningen, Department of Genetics, Groningen, The Netherlands; 6 Department of Cardiology, University of Groningen, University Medical Centre Groningen, Groningen, The Netherlands; 7 Department of Cardiology, Academic Medical Centre, University of Amsterdam, Amsterdam, The Netherlands; 8 Blueprint Genetics, Helsinki, Finland; 9 Durrer Centre for Cardiovascular Research, Netherlands Heart Institute, Utrecht, The Netherlands

Correction to: *Scientific Reports* 10.1038/s41598-019-39911-x, published online 11 March 2019

In the original version of this Article, the author J. Peter van Tintelen was incorrectly indexed. This error has now been corrected.

