# Supplementary material for: Relevance of Titin Missense and Non-Frameshifting Insertions/Deletions Variants in Dilated Cardiomyopathy
Source: Sci Rep. 2019 Mar 11;9:4093. doi: 10.1038/s41598-019-39911-x (PMC6412046; doi:10.1038/s41598-019-39911-x)
Supplement: Supplementary file 1 — Supplementary File [file 41598_2019_39911_MOESM1_ESM.docx]

**Relevance of Titin Missense and Non-Frameshifting Insertions/Deletions Variants in Dilated Cardiomyopathy**

**Short title:** Relevance of non-truncating titin variants in DCM

Oyediran Akinrinade^1,2^, Tiina Heliö, MD^3^, Ronald H. Lekanne Deprez^4^, Jan D.H. Jongbloed^5^, Ludolf G. Boven^5^, Maarten P van den Berg^6^, Yigal M. Pinto^7^, Tero-Pekka Alastalo^1,8^, Samuel Myllykangas^2,8^, Karin van Spaendonck-Zwarts, MD^4^, J. Peter van Tintelen^4,9^, Paul A. van der Zwaag^5^, Juha Koskenvuo^8^

^1^Children’s Hospital, Institute of Clinical Medicine, Helsinki University Central Hospital, University of Helsinki, Helsinki, Finland.

^2^Institute of Biomedicine, University of Helsinki, Helsinki, Finland.

^3^Heart and Lung Centre, Helsinki University Hospital and University of Helsinki, Helsinki, Finland.

^4^Department of Clinical Genetics, Academic Medical Centre, University of Amsterdam, Amsterdam, the Netherlands.

^5^University of Groningen, University Medical Centre Groningen, Department of Genetics, Groningen, the Netherlands.

^6^Department of Cardiology, University of Groningen, University Medical Centre Groningen, Groningen, the Netherlands.

^7^Department of Cardiology, Academic Medical Centre, University of Amsterdam, Amsterdam, the Netherlands.

^8^Blueprint Genetics, Helsinki, Finland

^9^Durrer Centre for Cardiovascular Research, Netherlands Heart Institute, Utrecht, the Netherlands.

**Table S1**. *TTN* missense variants in mutation positive vs. mutation negative DCM cohorts

|  | Total TTN missense Allele | | | |
| --- | --- | --- | --- | --- |
|  | Mutation positive (n=157) | Mutation negative (n=373) | OR | P-value |
| ***Raw variant (before assessment)*** | | | | |
| Unique variant count | 237 | 440 |  |  |
|  |  |  |  |  |
| ***After assessment using derived thresholds*** | | | | |
| Unique variant count | 26 | 70 |  |  |
| Number of individuals (n) | 29 | 71 |  |  |
| Prevalence (%) | 18.47 | 19.03 | 0.96 | 0.976 |
|  | | | | |
| ***Distribution by sarcomere domain, n (%)*** | | | | |
| Z-disc | - | 2 (0.54) | 0.47 | 1.000 |
| I-band | 13 (8.28) | 33 (8.85) | 0.93 | 1.000 |
| A-band | 17 (10.82) | 41 (10.99) | 0.98 | 1.000 |
| M-band | - | 2 (0.54) | 0.47 | 1.000 |
|  |  |  |  |  |
| ***After assessment using derived thresholds and pathogenicity prediction*** | | | | |
| Unique variant count | 9 | 22 |  |  |
| Number of individuals (n) | 11 | 25 |  |  |
| Prevalence (%) | 7.01 | 6.70 | 1.05 | 0.852 |
|  | | | | |
| ***Distribution by sarcomere domain, n (%)*** | | | | |
| Z-disc | - | 1 (0.27) | 0.79 | 1.000 |
| I-band | 4 (2.55) | 7 (1.88) | 1.37 | 0.739 |
| A-band | 7 (4.46) | 18 (4.83) | 0.92 | 1.000 |
| M-band | - | - | - | - |
